# Platelet-Rich Fibrin Can Neutralize Hydrogen Peroxide-Induced Cell Death in Gingival Fibroblasts

**DOI:** 10.3390/antiox9060560

**Published:** 2020-06-26

**Authors:** Zahra Kargarpour, Jila Nasirzade, Francesca Di Summa, Layla Panahipour, Richard J. Miron, Reinhard Gruber

**Affiliations:** 1 Department of Oral Biology, Medical University of Vienna, 1090 Vienna, Austria; zahra.kargarpooresfahani@meduniwien.ac.at (Z.K.); jila.nasirzaderajiri@meduniwien.ac.at (J.N.); francesca.disumma@studenti.unipr.it (F.D.S.); layla.panahipour@meduniwien.ac.at (L.P.); 2 Department of Periodontology, School of Dental Medicine, University of Bern, 3012 Bern, Switzerland; richard.miron@zmk.unibe.ch

**Keywords:** platelet-rich-fibrin, platelets, catalase, oxidative stress, acute toxicity, apoptosis, ulcer, wound healing, dentistry

## Abstract

Hydrogen peroxide is a damage signal at sites of chronic inflammation. The question arises whether platelet-rich fibrin (PRF), platelet-poor plasma (PPP), and the buffy coat can neutralize hydrogen peroxide toxicity and thereby counteract local oxidative stress. In the present study, gingival fibroblasts cells were exposed to hydrogen peroxide with and without lysates obtained from PRF membranes, PPP, heated PPP (75 °C for 10 min), and the buffy coat. Cell viability was examined by trypan blue staining, live-dead staining, and formazan crystal formation. Cell apoptosis was assessed by cleaved caspase-3 Western blot analysis. Reverse transcription-quantitative polymerase chain reaction (RT-PCR) was utilized to determine the impact of PRF lysates on the expression of catalase in fibroblasts. It was reported that lysates from PRF, PPP, and the buffy coat—but not heated PPP—abolished the hydrogen peroxide-induced toxicity in gingival fibroblasts. Necrosis was confirmed by a loss of membrane integrity and apoptosis was ruled out by the lack of cleavage of caspase-3. Aminotriazole, an inhibitor of catalase, reduced the cytoprotective activity of PRF lysates yet blocking of glutathione peroxidase by mercaptosuccinate did not show the same effect. PRF lysates had no impact on the expression of catalase in gingival fibroblasts. These findings suggest that PRF, PPP, and the buffy coat can neutralize hydrogen peroxide through the release of heat-sensitive catalase.

## 1. Introduction

Platelet-rich fibrin (PRF) is a fibrin-rich network that serves as a scaffold for bioactive molecules released from platelets being widely used in regenerative dentistry [1]. PRF has been shown to limit dimensional changes of the alveolar ridge post-extraction [2]. Furthermore, PRF has been utilized as an adjuvant for peri-implantitis therapy [3,4,5]. In general medicine, PRF has also been utilized to promote the healing and wound closure of chronic ulcers [6] and resurfacing of full-thickness burns [7]. The beneficial effects can be explained by the PRF intrinsic growth factors triggering cell responses of PRF including its ability to promote cell proliferation, migration, and differentiation [8,9]. Apart from its antimicrobial activity [10], PRF can also suppress inflammation [11] and osteoclastogenesis [12]. The complex effects of PRF presumably exceed those mediated simply by growth factors and we, therefore, proposed here that PRF may also support the local tissues by counteracting the reactive oxygen species and thereby minimizing oxidative stress.

Reactive oxygen species (ROS) are highly reactive molecules generated by local organisms and include hydrogen peroxide (H_2_O_2_), superoxide, and hydroxyl radical [13]. Under physiological conditions, hydrogen peroxide plays a pivotal role in cell growth and proliferation. Overproduction of hydrogen peroxide causes oxidative stress that may also lead to cellular damage and apoptotic cell death [14]. Hydrogen peroxide is neutralized by catalase (EC 1.11.1.6) and glutathione peroxidase (GPX; EC 1.11.1.9). Activated platelets liberate both catalase and glutathione peroxidase [15,16,17]. Also, erythrocytes accumulating next to the buffy coat layer at the transition of yellow and the red clot, are a rich source of catalase and glutathione peroxidase [18]. Catalase efficiently converts hydrogen peroxide into water and molecular oxygen. Glutathione peroxidase removes hydrogen peroxide at the expense of glutathione and requires selenium. Since platelets can consume hydrogen peroxide produced by neutrophils [19], it is plausible that platelets neutralize hydrogen peroxide and thereby protect cells involved in granulation tissue formation.

Recently, we have shown that supernatants of activated platelets efficiently neutralize toxic concentrations of hydrogen peroxide [20]. This neutralization, however, did not occur in the presence of the catalase inhibitor aminotriazole and was independent of glutathione peroxidase [17]. Similar findings were observed with erythrocytes. Inhibition of erythrocyte catalase abrogates their protective effect towards neutralizing high levels of exogenous hydrogen peroxide [18]. It is thus conceivable that PRF contains catalase. Catalase, however, is a heat-sensitive protein suggesting that pasteurization of platelet products may lose their activity [21]. This aspect is relevant as heating of liquid platelet-poor plasma (PPP) is applied to prolong the resorption of albumin gels [22,23] also when preparing injectable PRF consisting of autologous albumin gel and a liquid buffy coat (Alb-PRF) [24]. The aim of this study was, therefore, to examine whether standard PRF, standard and heated PPP, as well as the buffy coat layer, could neutralize the toxic concentration of exogenous hydrogen peroxide.

## 2. Materials and Methods

### 2.1. Cell Culture

Human gingiva was harvested during the extraction of impacted wisdom teeth from young and healthy patients who stayed anonymous and had given informed and written consent. A total of three strains of fibroblasts were established by explant cultures. Cells growing out from the gingiva explants were further expanded and stored in liquid nitrogen at low passage. Fibroblasts expanded for fewer than 10 passages were used for the experiments. Approval was obtained from the Ethics Committee of the Medical University of Vienna (EK NR 631/2007). A total of three strains of fibroblasts were established by explant cultures and fewer than 10 passages were used for the experiments. Gingival fibroblasts were grown and supplemented with 1% antibiotics (Sigma Aldrich, St. Louis, MO, USA) and 10% fetal calf serum (Bio&Sell GmbH, Nuremberg, Germany). The cells were exposed to the respective treatments for another 24 h under standard conditions at 37 °C, 5% CO_2_ and 95% humidity.

### 2.2. Preparation of Platelet-Rich Fibrin

PRF membranes were prepared after the approval of the ethics committee of the Medical University of Vienna (1644/2018) and volunteers signed informed consent. All experiments were performed in accordance with relevant guidelines and regulations. Venous blood was collected at the University Clinic of Dentistry from six non-fasting healthy volunteers (21G, Greiner Bio-One, Kremsmünster, Austria), each donating six plastic spray-coated silica tubes (BD Vacutainer^®^ Plymouth, UK) supporting spontaneous blood coagulation. PRF clots were produced by centrifugation at 400 *g* for 12 min utilizing a centrifuge device (Z 306 Hermle Universal Centrifuge, Wehingen, Germany) with universal swing-out rotors (146 mm at the max). The yellow PRF clot was separated from the remaining red clot and compressed between two layers of sterile gauzes to generate PRF membranes. PRF membranes were transferred into serum-free medium (1 cm PRF membrane/mL) and subjected to repeated freeze-thawing and sonication (Sonopuls 2000.2, Bandelin electronic, Berlin, Germany). After centrifugation at 15,000 *g* for 10 min (Eppendorf AG, Hamburg, Germany), the lysates were subjected to sterile filtration and stored at −20 °C prior to the analysis [11]. In indicated experiments, PRF membranes were transferred into a serum-free medium and the conditioned medium harvested after 24 and 72 h.

### 2.3. Preparation of Blood Fractions

For the preparation of albumin gels [24], venous blood was collected (21 G, Greiner Bio-One, Kremsmünster, Austria) in plastic tubes (“No Additive”, Greiner Bio-One GmbH, Kremsmünster, Austria) and centrifuged at 700 *g* for 8 min. The approximately 4 mL PPP, the 1 mL buffy coat layer, and the adjacent erythrocyte fraction were collected. To generate albumin gels (Alb-gel), PPP was heated at 75 °C for 10 min (Eppendorf, Thermomixer F1.5, Hamburg, Germany) and placed on crushed ice thereafter [25]. In another approach, 1 mL samples were pipetted precisely from the upper layer downward to consequently end up with 10 fractions. Each preparation was subjected to repeated freeze-thawing, transferred into an equal volume of serum-free medium and sonicated. After centrifugation at 15,000 *g* for 10 min, the lysates were subjected to sterile filtration and stored at −20 °C prior to the analysis.

### 2.4. Cell Viability Assay

For the viability assay, the various lysates were mixed with 3 mM H_2_O_2_ (Sigma Aldrich, St. Louis, MO, USA) and incubated for 10 min at room temperature. Catalase and glutathione peroxidase activities in PRF lysates were blocked by incubation with 100 mM aminotriazole (Sigma Aldrich, St. Louis, MO, USA) and 10 mM mercaptosuccinate (Sigma Aldrich, St. Louis, MO, USA), respectively. The cells were exposed to 10% of PRF lysates. After three hours of exposure, a cell viability assay was performed. For cell viability, MTT solution (Sigma Aldrich, St. Louis, MO, USA) at a final concentration of 0.5 mg/mL was added to each well of a microtiter plate and incubated for 2 h at 37 °C, 5% CO_2_ and 95% humidity. The medium was removed and the formazan crystals were solubilized with dimethyl sulphoxide (Sigma Aldrich, St. Louis, MO, USA). The optical density was measured at 570 nm. The data from independent experiments are presented as percentages of the optical density in the treatment groups normalized to the unstimulated control that was considered 100% viability regardless of the optical density.

### 2.5. Trypan Blue Staining and Live-Dead Staining

The lysates were incubated with 3 mM H_2_O_2_ for 10 min prior to cell stimulation for three hours. For testing the cellular membrane integrity, 0.4% trypan blue (Sigma Aldrich, St. Louis, MO, USA) diluted in PBS was added to each well and incubated for 10 min at room temperature. Trypan blue was discarded and the cells were examined by light microscopy. Cell viability was further confirmed using Live-dead staining assay kit according to the instructions of the manufacturer (Enzo Life Sciences, Inc., Lausanne, Switzerland).

### 2.6. Visualizing Bubble Assay and Bubble Microscopic Screening

Catalase rapidly converts hydrogen peroxide into water and molecular oxygen, and it is the oxygen that can be visualized using a bubble assay [26]. In brief, a solution containing 15% hydrogen peroxide and 0.5% Triton X-100 (Sigma Aldrich, St. Louis, MO, USA) was mixed with the equal volume of the various fractions or standard concentrations of bovine catalase in transparent round-bottomed test tubes (VWR International). The catalase-dependent production of oxygen was represented by the height of the foam generated. The experiments were carried out in triplicate. For rapid bubble screening, 100 µL of lysates were exposed to 10 µL of H_2_O_2_ on a glass slide and the reaction was recorded on the microscope by video and photograph.

### 2.7. Western Blot Analysis

Gingival fibroblasts were serum-starved overnight followed by three hours of stimulation with 100 µM etoposide, a cell-permeable inhibitor of topoisomerase II inhibitor (Calbiochem, Merck KGaA, Darmstadt, Germany), or 3 mM H_2_O_2_. Cell extracts containing protease inhibitors (Sigma Aldrich, St. Louis, MO, USA) were separated by SDS-polyacrylamide gel electrophoresis and transferred onto polyvinylidene difluoride membranes (Roche Diagnostic, Mannheim, Germany). The membranes were blocked with 5% dry milk and binding of the primary antibody cleaved caspase-3 (Cell Signaling Technology, Danvers, MA, USA) was detected with an appropriate secondary antibody directly labeled with peroxidase. Chemiluminescence signals were visualized with the ChemiDoc imaging system (Bio-Rad Laboratories, Inc. CA, USA).

### 2.8. Reverse Transcription Quantitative Polymerase Chain Reaction

Following stimulating the gingival fibroblast cells with 200 µM etoposide and 3 mM H_2_O_2_ for three hours, total RNA was isolated (ExtractMe, Blirt S.A., Gdańsk, Poland) and reverse transcription (RT) was performed (LabQ, Vienna, Austria). RT-PCR was done using the manufacturer’s instructions (LabQ, Vienna, Austria). Primer sequences were hBCL2-F: AGTACCTGAACCGGCACCT, hBCL2-R: GCCGTACAGTTCCACAAAGG; hGAPDH-F: AAGCCACATCGCTCAGACAC, hGAPDH-R: GCCCAATACGACCAAATCC. Relative gene expression was calculated with the delta-delta CT method using a software (CFX Maestro TM, BioRad, Hercules, CA, USA). Reactions were run in duplicates (Bio-Rad Laboratories, Inc. CA, USA).

### 2.9. Statistical Analysis

All experiments were performed three to five times. Bars show the mean and standard deviation of the cumulative data from the means of independent experiments. Statistical analysis was based on Mann–Whitney U test and Kruskal–Wallis test with Dunn multiple comparisons correction. The analysis was performed using statistical software. Significance was set at *p* < 0.05.

## 3. Results

### 3.1. PRF Lysates Rescue Cells from Acute Hydrogen Peroxide Toxicity

**Figure 1:**
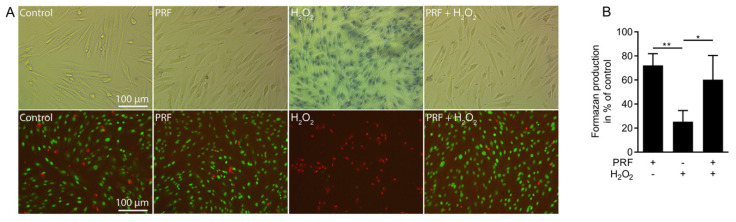
Platelet-rich fibrin (PRF) lysates rescue cells from acute hydrogen peroxide toxicity. Gingival fibroblasts (**A**) were exposed to 10% PRF lysates in the presence or absence of 3 mM H_2_O_2_ for 3 h. Necrotic cell death is shown by the uptake of trypan blue that however was not observed in the presence of PRF lysate. Live-dead staining was performed in gingival fibroblasts with viable cells appearing in green and dead cells in red. (**B**) Cell viability is represented by formazan production indicated in the percentage of unstimulated controls. The results from these experiments demonstrate that stimulation with 10% PRF lysates could neutralize the necrotic cell death caused by H_2_O_2_. Data represent the mean ± SD relative to the control. *n* = 4. * *p* < 0.05, ** *p* < 0.01, by two-tailed Mann–Whitney test.

First, we analyzed the effects of PRF lysates on human gingival fibroblasts treated with or without hydrogen peroxide. Hydrogen peroxide led to a rapid disruption of the cell membranes indicated by the uptake of trypan blue which was not observed in the presence of PRF lysates (Figure 1A). Similarly, PRF lysates inhibited the acute toxicity of hydrogen peroxide indicated by live-dead staining (Figure 1A). In support of this observation, PRF lysates prevented hydrogen peroxide-induced reduction of formazan crystals (Figure 1B). Together, these results suggest that PRF lysates attenuate hydrogen peroxide-induced acute necrotic cell death.

### 3.2. Hydrogen Peroxide Toxicity Occurs by Necrosis but Not by Apoptosis

**Figure 2:**
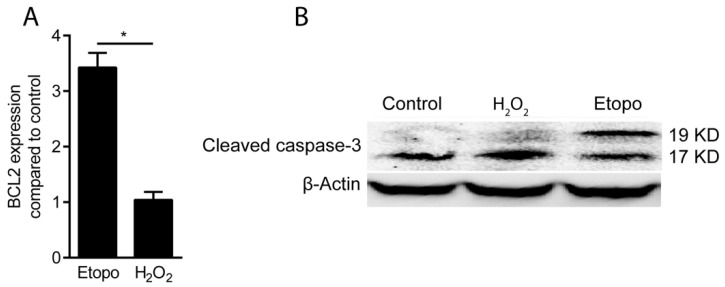
Hydrogen peroxide toxicity occurs by necrosis but not by apoptosis. Apoptosis of gingival fibroblasts was assessed by (**A**) evaluating the expression of the BCL2 gene and with (**B**) Western blot analysis of cleaved caspase-3 treated with H_2_O_2_ at 3 mM and etoposide at 200 µM for 3 h. It is indicated that H_2_O_2_ was not able to induce cleavage of caspase-3 while it was cleaved by apoptosis inducer, etoposide. All together it suggests that the decrease in cell viability is a consequence of necrosis rather than apoptosis (Etopo: Etoposide). *n* = 3. Data represent the mean ± SD. * *p* < 0.05, by two-tailed Mann–Whitney test.

To rule out the involvement of apoptosis, we utilized etoposide, a cell-permeable inhibitor of topoisomerase II inhibitor, to provoke apoptosis and cleavage of caspase-3 in gingival fibroblasts. Gene expression analysis showed that the level of the pro-apoptotic marker gene B cell lymphoma-2 (BCL2) increased in the presence of etoposide, but not with hydrogen peroxide (Figure 2A). This finding is further supported by Western blot analysis based on the endogenous levels of the large fragment (17/19 kDa) of activated caspase-3. We report here that etoposide, but not 3 mM hydrogen peroxide, increased the cleavage of caspase-3 into the two fragments (Figure 2B). These findings confirm that hydrogen peroxide-induced cell death is not mediated by apoptosis. These observations suggest that the sharp decrease in cell viability is a consequence of necrosis rather than apoptosis.

### 3.3. PRF Conditioned Medium Protects Cells from Necrotic Cell Death

**Figure 3:**
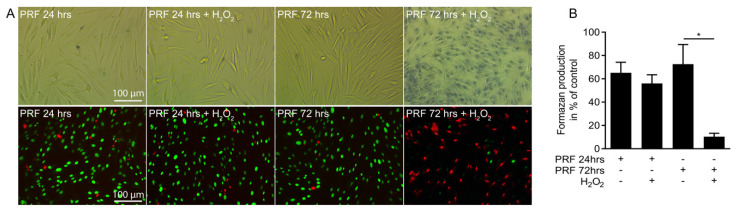
PRF conditioned medium protects cells from necrotic cell death. Gingival fibroblasts (**A**) were exposed to 10% PRF conditioned medium collected at 24 and 72 hours in the presence or absence of 3 mM H_2_O_2_ for 3 hours. Necrotic cell death is shown by the uptake of trypan blue. Live-dead staining was performed in gingival fibroblasts with viable cells appearing in green and dead cells in red. (**B**) Cell viability is represented by formazan production indicated in percentage of unstimulated controls. The results from these experiments demonstrated that stimulation with 10% PRF conditioned media obtained at 24 hours preserves the cells from necrosis induced by H_2_O_2_. Data represent the mean ± SD relative to the control. *n* = 4. Data represent the mean ± SD. * *p* < 0.05, by two-tailed Mann-Whitney test.

Next, we investigated whether PRF conditioned medium could neutralize the hydrogen peroxide toxicity. PRF conditioned medium obtained after 24 h protected gingival fibroblasts from the acute toxicity of hydrogen peroxide indicated by the uptake of trypan blue and live-dead staining (Figure 3A), and the formation of formazan crystals (Figure 3B). PRF conditioned medium obtained after 72 h however, failed to protect the cells from cell death. These findings indicate that conditioned medium harvested from fresh PRF membranes can suppress hydrogen peroxide-induced cell death.

### 3.4. Blocking Catalase Attenuates the Activity of PRF Lysate in Preventing the Cells from Cell Death

**Figure 4:**
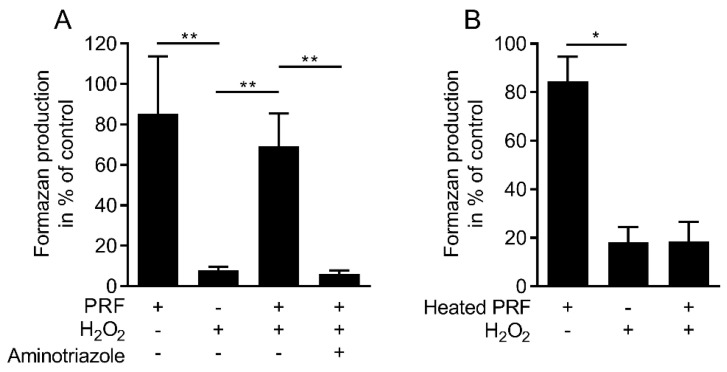
Blocking catalase attenuates the activity of PRF lysate in preventing cell death. Gingival fibroblasts (**A**) were exposed to 10% PRF in the presence or absence of 3 mM H_2_O_2_ and 100 mM catalase inhibitor, aminotriazole. Cell viability is measured by the conversion of MTT into formazan crystals. (**B**) MTT assay also demonstrated that heated PRF is not able to neutralize H_2_O_2_. *n* = 3–5. Data represent the mean ± SD. * *p* < 0.05, ** *p* < 0.01, by two-tailed Mann–Whitney test.

To examine whether the protective activity of PRF lysate involved catalase, the catalase inhibitor aminotriazole was used. Aminotriazole (100 mM) abolished the cytoprotective activity of PRF lysates (Figure 4A). Similar to purified platelets [17], blocking of glutathione peroxidase by mercaptosuccinate failed to reduce the cytoprotective activity of PRF lysates (data not shown). Considering that catalase is heat-sensitive [26], PRF lysates heated at 95 °C for 30 min lost the ability to neutralize hydrogen peroxide (Figure 4B). Based on a standard curve, undiluted PRF lysates contain approximately 30 unit/mg of catalase (Supplementary Figure S1). Taken together, these data suggest that the protective activity of PRF lysates is mediated by catalase.

### 3.5. PRF Capacity in Neutralizing Hydrogen Peroxide Is Limited

**Figure 5:**
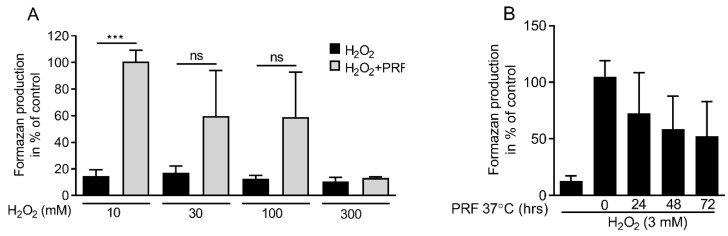
PRF capacity in neutralizing hydrogen peroxide is dose-dependent. (**A**) Gingival fibroblast cells were exposed to PRF lysates in the presence of various doses of H_2_O_2_ (10, 30, 100, 300 mM). MTT results confirmed cell viability in the presence of PRF lysates for all the groups except 300 mM. (**B**) The neutralizing capacity of PRF lysates incubated at 37 °C for 24, 48, and 72 h were also tested in the presence of 3 mM H_2_O_2_. The results suggest that catalase activity was decreased in a time-dependent manner. *n* = 3–5. Data represent the mean ± SD. *** *p* < 0.001 and ns = not significant, by two-tailed Mann–Whitney test.

To figure out the concentration where PRF lost the ability to neutralize hydrogen peroxide, a dose-response experiment was carried out. Gingival fibroblasts were exposed to different concentrations of hydrogen peroxide in the presence or absence of 10% PRF lysates. The formazan production indicated that 10, 30, and 100 mM hydrogen peroxide, but not 300 mM were neutralized by 10% PRF (Figure 5A). To measure the stability of catalase, PRF lysates were stored at 37 °C for 24, 48, and 72 h. The results demonstrated that catalase in PRF lysates kept its activity over time (Figure 5B). Thus, PRF lysates could effectively neutralize high amounts of hydrogen peroxide and maintain the activity upon storage.

### 3.6. Regular PPP, Buffy Coat and Red Blood Clot but Not Albumin Gel Neutralize Hydrogen Peroxide

**Figure 6:**
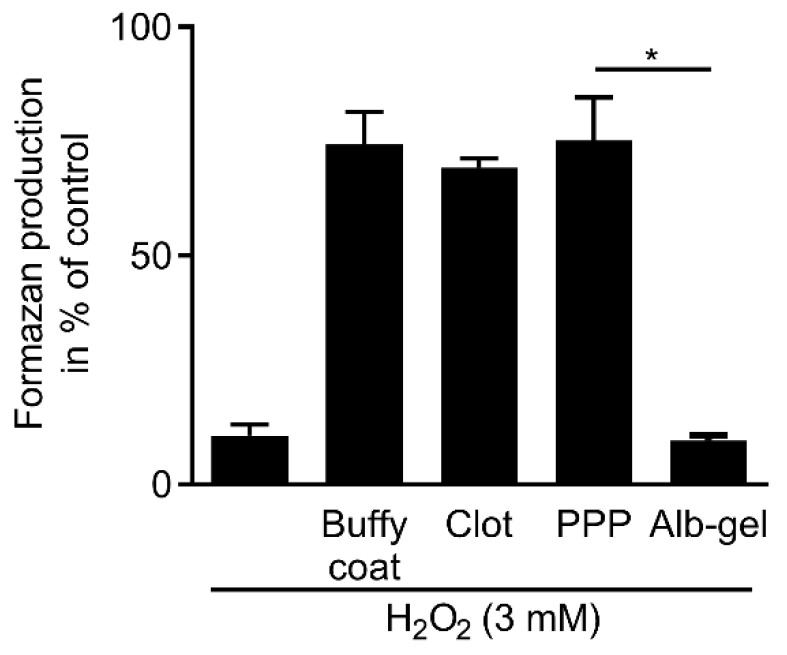
Buffy coat layer and blood clot but not the albumin gel neutralizes H_2_O_2_ cytotoxicity. Gingival fibroblast cells were incubated with a buffy coat layer, blood clot, PPP, and Alb-gel (heated PPP) in the presence of 3 mM H_2_O_2_. MTT results showed that heating at 75 °C for 10 min can suppress catalase neutralizing activity while catalase remains active in all the other components. *n* = 3. Data represent the mean ± SD. * *p* < 0.05, by two-tailed Mann–Whitney test.

The next experiments were based on the heat sensitivity of catalase [27] based on the recent introduction of an injectable PRF mixture where the PPP is heated at 75 °C for 10 min to create denatured albumin (albumin gel) [24]. Viability assays confirmed that lysates of heated PPP lose the catalase activity, while lysates from regular PPP, the buffy coat layer and the remaining red clot neutralize hydrogen peroxide. These data support the assumption that the catalase activity of the injectable PRF mixture is present in the PPP and buffy coat layer but not the albumin gel due to heating (Figure 6). The findings were further confirmed by visualizing and quantifying bubble assay and microscopic bubble screening (Supplementary Figures S2–S3 and Table S1). In this assay, the buffy coat layer and the remaining red clot fraction cause significantly more oxygen than the respective PPP lysates supporting the high catalase activity of erythrocytes [18].

## 4. Discussion

Even though PRF is extensively used to support wound healing and bone regeneration, and was recently proposed for facial esthetics [28,29], the underlying molecular and cellular mechanisms remain unclear. The beneficial effects of PRF in alveolar ridge preservation, keratinized tissue augmentation [3], and healing of chronic ulcers [3] might be attributed to its inhibitory effect on osteoclastogenesis [12] along with its pro-angiogenic [30], pro-migratory, proliferative [20], and anti-inflammatory effect [8,11]. Based on the data presented here, PRF might also fight tissue damage and support tissue regeneration by serving as a source of catalase, the evolutionary highly conserved and still mysterious enzyme effectively neutralizing reactive hydrogen peroxide [31]. The main finding of this report is that it is particular the buffy coat and the adjacent red blood layer that showed the strongest catalase activity compared to PPP. Moreover, preparing albumin gels by heating PPP greatly abolishes the catalase activity.

Our findings are in agreement with previous studies showing that activated platelets can efficiently neutralize toxic amounts of hydrogen peroxide by the release of catalase [17]. Consistent with previous reports, the inhibition of the catalase activity by using aminotriazole hindered PRF lysate to exert its protective effect on cells against a high concentration of hydrogen peroxide [17]. The same is true for erythrocytes being a potent source of catalase [18]. The results presented here are also aligned with previous research indicating that hydrogen peroxide at toxic concentrations provokes necrosis and not apoptosis [17]. Moreover, it can be ruled out that the neutralization of the acute toxicity of hydrogen peroxide is not caused by the catalase produced by the gingival fibroblasts. In support of the general understanding of catalase is also the heat sensitivity and that pasteurization of platelet products may decrease or even abolish its activity [21]. Taken together, these findings indicate that PRF, similar to activated platelets, can neutralize hydrogen peroxide-induced necrosis by the release of catalase.

The clinical relevance of the in vitro observations leaves room for speculations. Even though it remains unclear whether platelets are the exclusive source of catalase, these findings provide new perspectives to understand how PRF exerts its beneficial effects in vivo [5]. High amounts of native catalase are present in the healthy skin organ [32] but not in situations where wound healing is impaired by the lack of catalase including skin diseases, such as vitiligo [33], psoriasis [34], and presumably also in refractory leg ulcers [6]. PRF and its respective membrane, and particular the buffy coat layer including parts of the erythrocyte fraction could become a rich source of catalase to support wound healing and may replace or complement traditional clinical strategies where catalase and mimetics are applied for the treatment of skin diseases and wound injury [33,35]. Care should be taken not to pasteurize PRF in the context of virus inactivation and possible allogeneic transplantation, as catalase is temperature-sensitive. Therefore, the clinical application of PRF might be particularly effective at sites of chronic inflammation with low levels of catalase where excessive reactive oxygen species and hydrogen peroxide are not neutralized thereby impairing wound healing.

Considering that chronic inflammatory diseases such as periodontitis are characterized by oxidative stress signals [36] and various reactive oxygen species are liberated under inflammatory conditions [37], PRF might not only exert its anti-inflammatory effect via macrophage polarization [11] but also through neutralization of the hydrogen peroxide-induced inflammation [38]. Catalase might be involved in explaining the positive effects of PRF in refractory skin ulcers [6]. For example, the local application of catalase on thermal skin injury helped to reduce the lesion size in rats [39]. The present data are also relevant to consider heating when preparing an injectable PRF mixture consisting of autologous albumin gel and liquid platelet-rich fibrin. Heating the platelet-poor plasma layer at 75 °C for 10 min not only changes the resorption properties but also abolishes catalase as well as the glutathione peroxidase activity [24]. It is thus the buffy coat layer keeping the catalase activity of Alb-PRF [24].

A number of limitations should be considered when interpreting the results of this study. Firstly, the origin of the catalase remains unclear but considering that platelets [16,17] and not neutrophils are a major source of catalase [40], the cellular origin can be proposed. Moreover, the number of cells other than platelets in PRF produced at 400 g is rather low [41]. Second, neutrophils release other reactive oxygen species than hydrogen peroxide that may damage cells during overshooting inflammation. Our findings further support the role of the red clot being a rich source of catalase, likely because of the erythrocytes [18]. Future research should also investigate the neutralizing capacity of PRF lysate on reactive oxygen species such as superoxide, hydroxyl radical, singlet oxygen, and alpha-oxygen. Third, platelets also release other detoxifying enzymes that are embedded in the fibrin-rich matrix, therefore, PRF research should not be restricted to the catalase activity. Forth, platelet-rich plasma is also applied clinically covering some of the same indications as PRF, and presumably also contains catalase. This, however, requires to be confirmed experimentally. Finally, it remains unclear whether the catalase can explain some of the clinical outcomes of PRF, not only in the field of dentistry but also with respect particularly to the healing of ulcers. 

## 5. Conclusions

Taken together, these results suggest that PRF can neutralize cytotoxic amounts of hydrogen peroxide through the release of catalase. Heating, however, can diminish the catalase activity in PRF.

## Supplementary Materials

The following are available online at https://www.mdpi.com/2076-3921/9/6/560/s1, Figure S1: Catalase activity measured by MTT assay, Figure S2: Visualizing bubble assay for catalase activity was analyzed based on the height of oxygen-forming foam, Figure S3: Microscopic illustration for bubbles confirmed that heating inactivates catalase, Table S1: Quantitative activity of catalase for the standard and the samples, Video S1: Microscopic illustration for bubbles by catalase.
