# Supplementary material for: Platelet-Rich Fibrin Can Neutralize Hydrogen Peroxide-Induced Cell Death in Gingival Fibroblasts
Source: Antioxidants (Basel). 2020 Jun 26;9(6):560. doi: 10.3390/antiox9060560 (PMC7346145; doi:10.3390/antiox9060560)
Supplement: Supplementary file 1 [file antioxidants-09-00560-s001.zip › antioxidants-842997/supplementary antioxidants-842997.docx]

**Supplement Figures**

**
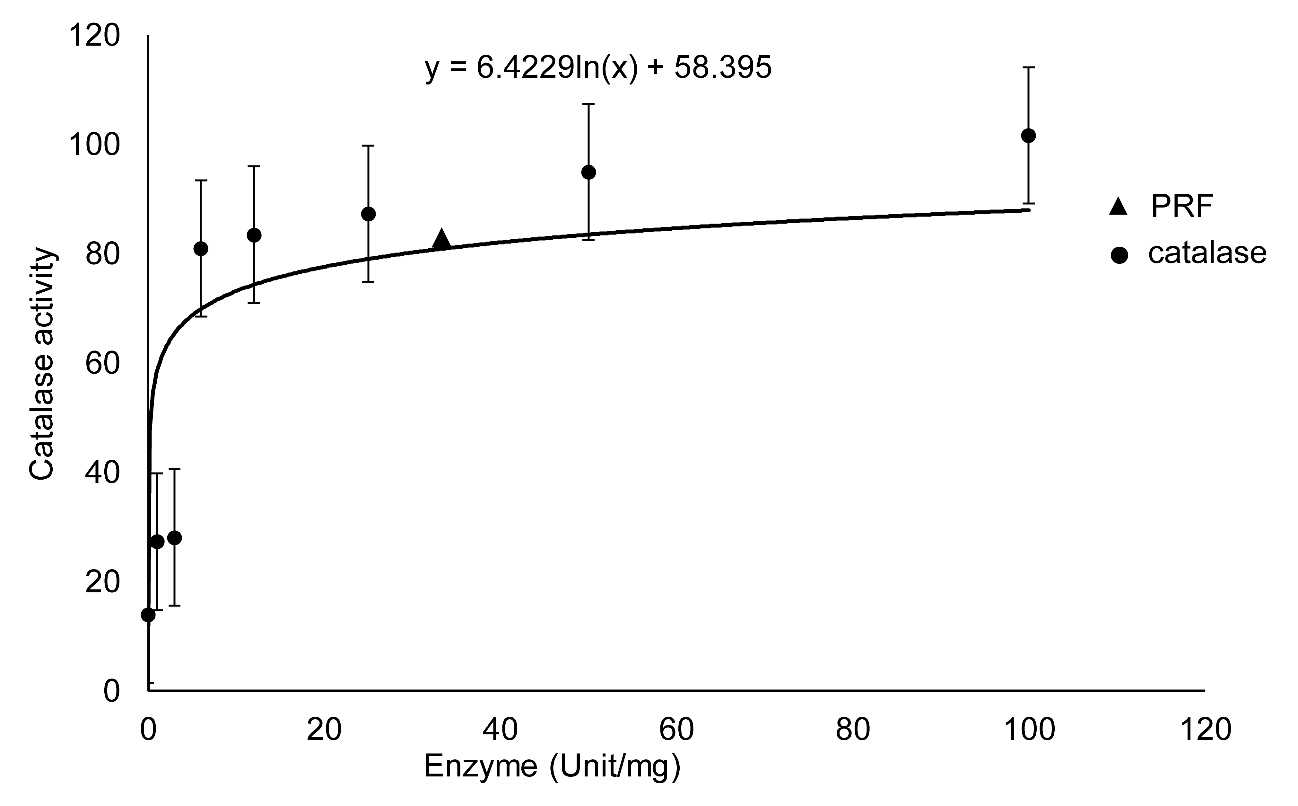
**

*Supplementary Figure 1. Catalase activity measured by MTT assay.*

Gingival fibroblasts were exposed to defined units of catalase and 3mM H_2_O_2_.The results were evaluated by MTT assay. Catalase activity was the optical density measured by MTT assay to be used as calibration curve. Each point represents the average optical density of three independent experiments. The function driven from the plot is y=6.4229ln(x)+58.395. Consistently, the catalase activity in 10% PRF lysates was evaluated based on the average optical density obtained from three independent MTT assays in the presence of PRF and H_2_O_2_. Based on this setting the catalase unit in the 10% PRF lysates was estimated to be 30 units.


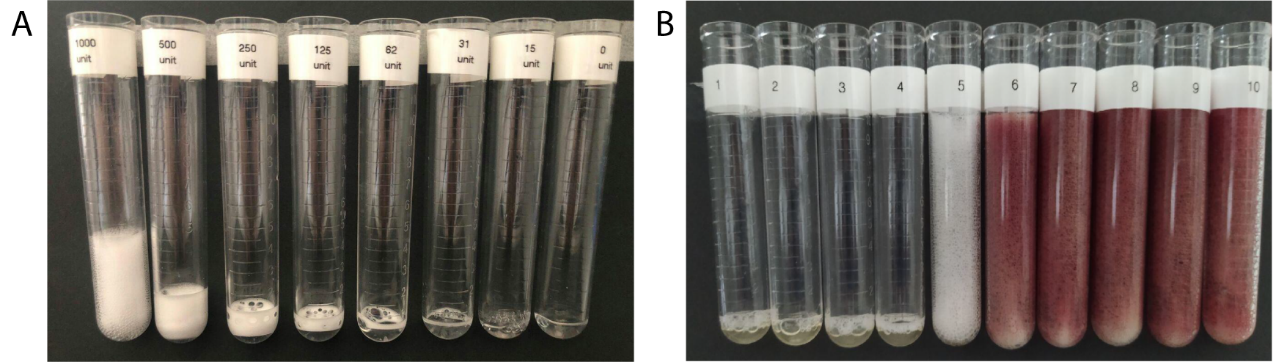


*Supplementary Figure 2.* *Visualizing bubble assay for catalase activity was analyzed based on the height of oxygen-forming foam.* (A) The image shows different heights of foam developed in each test tube following the reaction of catalase designated by the defined units of enzyme with H_2_O_2_. (B) The 1-ml fractions of injectable liquid PRF were also exposed to H_2_O_2_ in the transparent plastic tubes. It is indicated that buffy coat layer and all the clot fractions (fractions number 5 to 10) has a high catalase activity compared to the first 4 fractions representative for PPP.


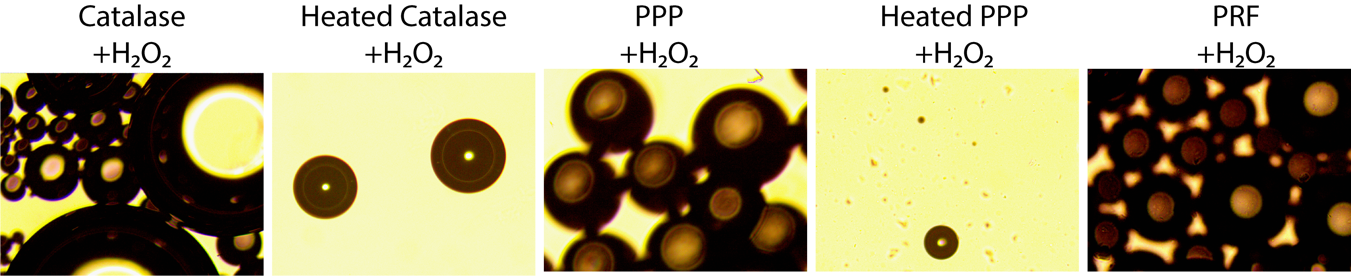


*Supplementary Figure 3. Microscopic illustration for bubbles confirmed that heating inactivates catalase.* H_2_O_2_ (10 mM) was added to catalase, heated catalase, PPP, heated PPP (Alb-gel) and PRF. Subsequently, the reaction was screened under the microscope. The amount of appeared bubbles are representative for catalase activity. The figure shows that heating catalase and PPP at 75˚C for 10 minutes can significantly suppress enzymatic activity.

| 1. Catalase activity (unit) | 0 | 15 | 31 | 62 | 125 | 250 | 500 | 1000 |
| --- | --- | --- | --- | --- | --- | --- | --- | --- |
| Exp 1  Exp 2  Exp 3 | 0 | 2 | 4 | 6 | 6 | 10 | 17 | 38 |
|  | 0 | 3 | 4 | 5 | 6 | 8 | 19 | 39 |
|  | 0 | 3 | 5 | 5 | 7 | 9 | 15 | 37 |

| 1. PRF ml fractions | 1 | 2 | 3 | 4 | 5 | 6 | 7 | 8 | 9 | 10 |
| --- | --- | --- | --- | --- | --- | --- | --- | --- | --- | --- |
| Exp 1  Exp 2  Exp 3 | 7 | 7 | 6 | 7 | 16 | 12 | 18 | 18 | 18 | 18 |
|  | 5 | 5 | 6 | 4 | 17 | 17 | 17 | 18 | 17 | 18 |
|  | 6 | 5 | 5 | 4 | 18 | 19 | 19 | 19 | 18 | 17 |

*Supplementary Table 1. Quantitative activity of catalase for the standard and the samples.*

The numbers indicate the height of foam in mm generated after mixing catalase or the samples, Triton X-100, and H_2_O_2_. The reactions were done in triplicate and the numbers are measured from the baseline which is the initiating height. The results are represented for (A) catalase standards and (B) the samples. Defined units of catalase were applied to plot the catalase standard curve and the respective calibration curve was plotted using the defined units of catalase activity (y=0.0352x+1.5165, r=0.9861).

SEE ATTACHMENT

*Supplementary video 1. Microscopic video records of bubbles by catalase.* H_2_O_2_ (10 mM) was added to (A) catalase, (B) heated catalase, (C) PPP, (D) heated PPP (Alb-gel) and (E) PRF lysates and reactions were subsequently recorded under the microscope. The bubbles represent catalase activity. The videos show that heating catalase and PPP at 75˚C for 10 minutes can significantly suppress bubble formation.
