# Supplementary figures and images for: Platelet-Rich Fibrin Can Neutralize Hydrogen Peroxide-Induced Cell Death in Gingival Fibroblasts
Source: Antioxidants (Basel). 2020 Jun 26;9(6):560. doi: 10.3390/antiox9060560 (PMC7346145; doi:10.3390/antiox9060560)

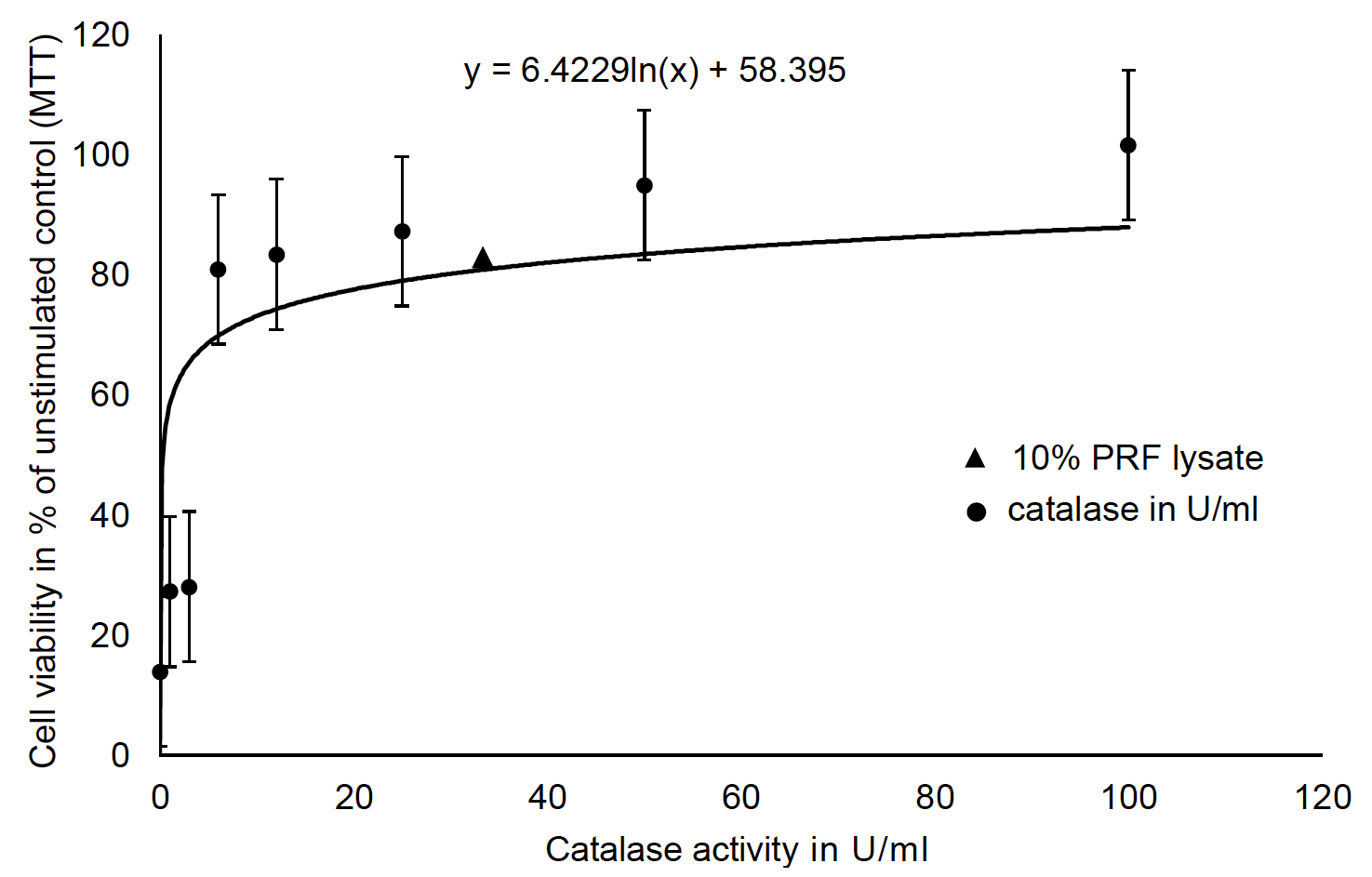

Supplement: Supplementary file 1 [file antioxidants-09-00560-s001.zip › antioxidants-842997/Supplementary files/Supplement Figure 1.tif]

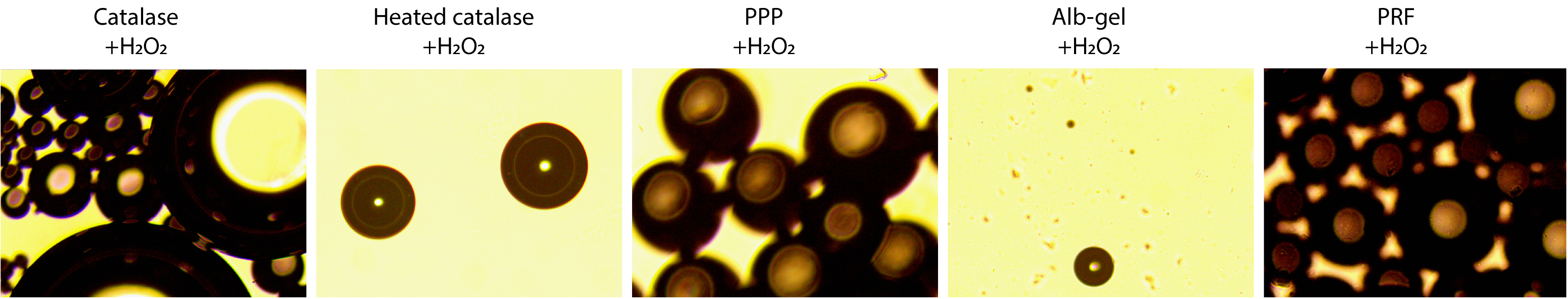

Supplement: Supplementary file 1 [file antioxidants-09-00560-s001.zip › antioxidants-842997/Supplementary files/Supplement Figure 3.tif]
